# 2-Amino-1-methyl-4-oxo-4,5-dihydro-1*H*-imidazol-3-ium chloride

**DOI:** 10.1107/S1600536812027080

**Published:** 2012-06-23

**Authors:** Masoumeh Tabatabaee, Mahboubeh A. Sharif, Michal Dušek, Michaela Pojarová

**Affiliations:** a Department of Chemistry, Yazd Branch, Islamic Azad University, Yazd, Iran; b Department of Chemistry, Qom Branch, Islamic Azad University, Qom, Iran; c Institute of Physics ASCR, v.v.i., Na Slovance 2, 182 21 Praha 8, Czech Republic

## Abstract

In the crystal structure of the title compound, C_4_H_8_N_3_O^+^·Cl^−^, N—H⋯Cl hydrogen bonds link the components into chains along [010]. In addition, weak C—H⋯Cl hydrogen bonds link the chains into a two-dimensional network perpendicular to (001).

## Related literature

For creatinine (2-amino-1-methyl-5H-imidazol-4-one), which is used in the synthesis of some 1:1 proton-transfer compounds, see; Moghimi *et al.* (2004 ▶); Soleimannejad *et al.* (2005 ▶). For related structures, see: Tabatabaee *et al.* (2007 ▶); Bujak & Zaleski (2002 ▶); Tabatabaee, Abbasi *et al.* (2011 ▶); Tabatabaee, Tahriri *et al.* (2011 ▶, 2012 ▶); Tabatabaee, Adineh *et al.* (2012 ▶). For background information on weak C—H⋯Cl hydrogen bonds, see: Freytag & Jones (2000 ▶); Taylor & Kennard (1982 ▶).
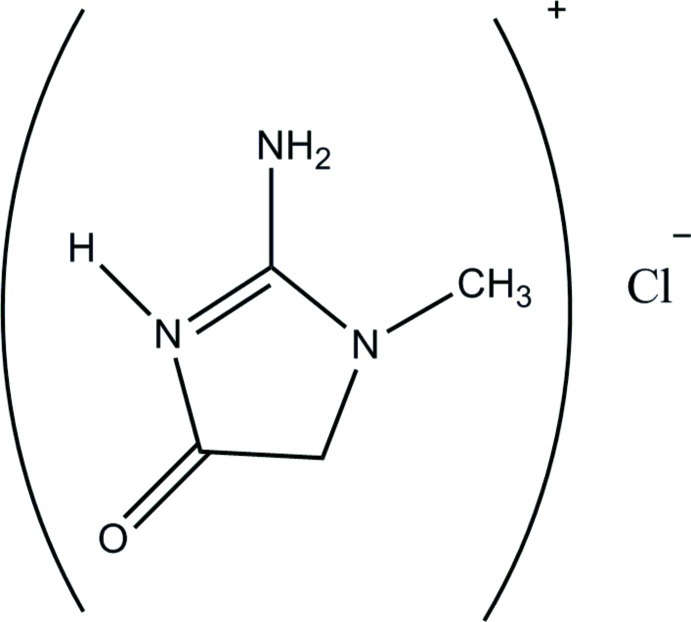


## Experimental

### 

#### Crystal data

C_4_H_8_N_3_O^+^·Cl^−^
*M* *_r_* = 149.58
Monoclinic, 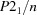

*a* = 8.4617 (2) Å
*b* = 7.7073 (2) Å
*c* = 10.2215 (3) Å
β = 98.369 (2)°
*V* = 659.52 (3) Å^3^
*Z* = 4
Cu *K*α radiation
μ = 4.51 mm^−1^
*T* = 120 K
0.57 × 0.35 × 0.15 mm

#### Data collection

Oxford Diffraction Xcalibur Atlas Gemini ultra diffractometer
Absorption correction: multi-scan (*CrysAlis PRO*; Oxford Diffraction, 2010 ▶) *T*_min_ = 0.509, *T*_max_ = 1.000
5373 measured reflections
1167 independent reflections
1158 reflections with *I* > 2σ(*I*)
*R*_int_ = 0.023

#### Refinement

*R*[*F* ^2^ > 2σ(*F* ^2^)] = 0.026
*wR*(*F* ^2^) = 0.074
*S* = 1.08
1167 reflections
83 parameters
H-atom parameters constrained
Δρ_max_ = 0.28 e Å^−3^
Δρ_min_ = −0.18 e Å^−3^

### 

Data collection: *CrysAlis CCD* (Oxford Diffraction, 2007 ▶); cell refinement: *CrysAlis CCD* data reduction: *CrysAlis RED* (Oxford Diffraction, 2007 ▶); program(s) used to solve structure: *SHELXS97* (Sheldrick, 2008 ▶); program(s) used to refine structure: *SHELXL97* (Sheldrick, 2008 ▶); molecular graphics: *PLATON* (Spek, 2009 ▶) and *DIAMOND* (Brandenburg, 1999 ▶); software used to prepare material for publication: *publCIF* (Westrip, 2010 ▶).

## Supplementary Material

Crystal structure: contains datablock(s) I, global. DOI: 10.1107/S1600536812027080/lh5487sup1.cif

Structure factors: contains datablock(s) I. DOI: 10.1107/S1600536812027080/lh5487Isup2.hkl

Supplementary material file. DOI: 10.1107/S1600536812027080/lh5487Isup3.cml

Additional supplementary materials:  crystallographic information; 3D view; checkCIF report

## Figures and Tables

**Table 1:** Hydrogen-bond geometry (Å, °)

*D*—H⋯*A*	*D*—H	H⋯*A*	*D*⋯*A*	*D*—H⋯*A*
N1—H1⋯Cl1^i^	0.86	2.42	3.2714 (12)	169
N1—H2⋯Cl1^ii^	0.86	2.32	3.1506 (12)	163
N2—H3⋯Cl1	0.89	2.31	3.1808 (11)	165
C2—H4⋯Cl1^iii^	0.97	2.69	3.6271 (14)	162
C4—H8⋯Cl1^i^	0.96	2.77	3.7241 (13)	175

Symmetry codes: (i) 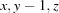; (ii) 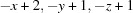; (iii) 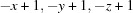.

**Fig. 1.**
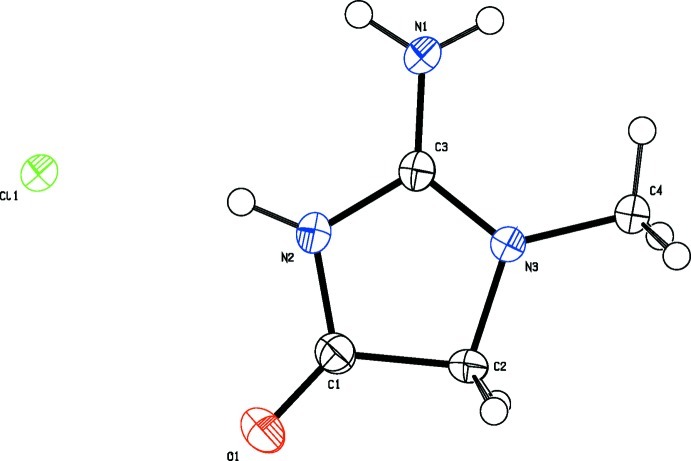
The molecular structure of (I). Displacement ellipsoids are drawn at the 50% probability.

**Fig. 2.**
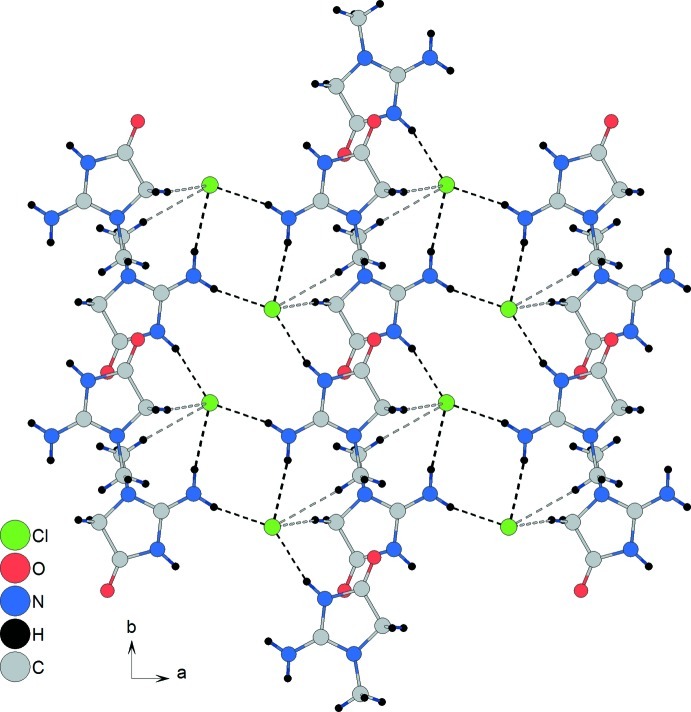
Part of the crystal structure with N—H···Cl hydrogen bonds shown as black dashed lines and weak C—H···Cl hydrogen bonds shown as grey dashed lines.

## supplementary crystallographic information

### Comment

In continuation of our research to synthesize transition metal complexes with dicarboxylic acids (especially pyridine-2,6-dicarboxilic acid) in the presence of some amino compounds (Tabatabaee, Abbasi *et al.*, 2011; Tabatabaee, Tahriri *et al.*, 2011; Tabatabaee, Tahriri *et al.*, 2012; Tabatabaee, Adineh *et al.*, 2012), the reaction of zirconium tetrachloride, with pyridine-2,6-dicarboxilic acid in the presence of creatinine was performed. The title compound (I) was fortuitously obtained as a result of this reaction. Creatinine has previously been used as a proton acceptor in the synthesis of some 1:1 proton-transfer compounds (Moghimi *et al.*, 2004; Soleimannejad *et al.*, 2005).

The molecular structure of (I) is shown in Fig. 1. During the reaction a proton was transferred to the ring N atom of the creatinine (2-Amino-1-methyl-5H-imidazol-4-one) molecule. In (I) the C3—N1 bond [1.3094 (18) Å] and C3—N2 bond [1.3647 (17) Å] can be compared to the C═N bond [1.3108 (18) Å] and C—N bond [1.3612 (17) Å] in the reported proton transfer compound, bis(creatininium)2,5-dicarboxybenzene-1,4-dicarboxylate (Tabatabaee *et al.*, 2007).

In the crystal, intermolecular N—H···Cl hydrogen bonds link the components into one-dimensional chains along [010]. In addition, weak intermolecular C—H···Cl hydrogen bonds link one-dimensional-chains into a two-dimensional network perpendicular to (001) (Fig. 2). When compared with the crystal structure of 1,2,4-triazolium chloride (Bujak & Zaleski 2002), the N—H···Cl interactions are weaker in the present structure while C—H···Cl interactions are similar. For the weak intermolecular hydrogen bonds the C—H···Cl angles are in the range of those previously reported (Freytag & Jones, 2000; Taylor & Kennard, 1982).

### Experimental

An aqueous solution of ZrCl_4_, (0.233 g, 1 mmol) in water (10 ml) was added to a stirring solution of (20 ml) pyridine-2,6-dicarboxylic acid (0.167 g, 1 mmol) and creatinine (0.113 g, 1 mmol). The reaction mixture was stirred at 298K for 4 h. The resulting solid residue was filtered and the colorless crystals of the title compound were obtained after few days at 277K from mother liquor.

### Refinement

H atoms bonded to C atoms were included in calculated positions with C—H = 0.96 and 0.97Å and with U_iso_(H) = 1.5U_eq_(C). H atoms bonded to N atom were included with N—H 0.86 amd 0.89Å and with U_iso_(H) = 1.5U_eq_(N).

### Crystal data

**Table:**

C_4_H_8_N_3_O^+^·Cl^−^	*F*(000) = 312
*M**_r_* = 149.58	*D*_x_ = 1.507 Mg m^−^^3^
Monoclinic, *P*2_1_/*n*	Cu *K*α radiation, λ = 1.54178 Å
Hall symbol: -P 2yn	Cell parameters from 5086 reflections
*a* = 8.4617 (2) Å	θ = 4.4–66.9°
*b* = 7.7073 (2) Å	µ = 4.51 mm^−^^1^
*c* = 10.2215 (3) Å	*T* = 120 K
β = 98.369 (2)°	Plate, colourless
*V* = 659.52 (3) Å^3^	0.57 × 0.35 × 0.15 mm
*Z* = 4	

### Data collection

**Table:**

Oxford Diffraction Xcalibur Atlas Gemini ultra diffractometer	1167 independent reflections
Radiation source: Enhance Ultra (Cu) X-ray Source	1158 reflections with *I* > 2σ(*I*)
Mirror monochromator	*R*_int_ = 0.023
Detector resolution: 10.3784 pixels mm^-1^	θ_max_ = 67.0°, θ_min_ = 6.4°
Rotation method data acquisition using ω scans	*h* = −10→9
Absorption correction: multi-scan (*CrysAlis PRO*; Oxford Diffraction, 2010)	*k* = −9→9
*T*_min_ = 0.509, *T*_max_ = 1.000	*l* = −12→11
5373 measured reflections	

### Refinement

**Table:**

Refinement on *F*^2^	Primary atom site location: structure-invariant direct methods
Least-squares matrix: full	Secondary atom site location: difference Fourier map
*R*[*F*^2^ > 2σ(*F*^2^)] = 0.026	Hydrogen site location: inferred from neighbouring sites
*wR*(*F*^2^) = 0.074	H-atom parameters constrained
*S* = 1.08	*w* = 1/[σ^2^(*F*_o_^2^) + (0.0471*P*)^2^ + 0.1996*P*] where *P* = (*F*_o_^2^ + 2*F*_c_^2^)/3
1167 reflections	(Δ/σ)_max_ < 0.001
83 parameters	Δρ_max_ = 0.28 e Å^−^^3^
0 restraints	Δρ_min_ = −0.18 e Å^−^^3^

### Special details

**Table:**

Geometry. All e.s.d.'s (except the e.s.d. in the dihedral angle between two l.s. planes) are estimated using the full covariance matrix. The cell e.s.d.'s are taken into account individually in the estimation of e.s.d.'s in distances, angles and torsion angles; correlations between e.s.d.'s in cell parameters are only used when they are defined by crystal symmetry. An approximate (isotropic) treatment of cell e.s.d.'s is used for estimating e.s.d.'s involving l.s. planes.
Refinement. Refinement of *F*^2^ against ALL reflections. The weighted *R*-factor *wR* and goodness of fit *S* are based on *F*^2^, conventional *R*-factors *R* are based on *F*, with *F* set to zero for negative *F*^2^. The threshold expression of *F*^2^ > σ(*F*^2^) is used only for calculating *R*-factors(gt) *etc*. and is not relevant to the choice of reflections for refinement. *R*-factors based on *F*^2^ are statistically about twice as large as those based on *F*, and *R*- factors based on ALL data will be even larger. The H atoms were all located in a difference map, but those attached to carbon atoms and the nitrogen atom in amino group were repositioned geometrically. The H atoms were initially refined with soft restraints on the bond lengths and angles to regularize their geometry (C—H in the range 0.93–0.98, N—H in the range 0.86–0.89 N—H to 0.86 O—H = 0.82 Å) and *U*_iso_(H) (in the range 1.2 times *U*_eq_ of the parent atom). The distance between hydrogen atom H3 and N2 was left unrestrained.

### Fractional atomic coordinates and isotropic or equivalent isotropic displacement parameters (Å^2^)

**Table:**

	*x*	*y*	*z*	*U*_iso_*/*U*_eq_	
Cl1	0.86814 (3)	0.71590 (4)	0.45236 (3)	0.02046 (16)	
O1	0.43618 (13)	0.57647 (13)	0.32081 (12)	0.0358 (3)	
N1	0.79940 (13)	0.13245 (15)	0.41934 (11)	0.0238 (3)	
H1	0.8035	0.0210	0.4217	0.029*	
H2	0.8847	0.1923	0.4421	0.029*	
N2	0.64629 (13)	0.38708 (14)	0.37714 (11)	0.0217 (3)	
H3	0.7227	0.4646	0.4031	0.026*	
C1	0.48933 (16)	0.43236 (18)	0.33205 (13)	0.0234 (3)	
C2	0.40103 (16)	0.26312 (17)	0.30246 (14)	0.0211 (3)	
H4	0.3128	0.2525	0.3529	0.025*	
H5	0.3606	0.2526	0.2090	0.025*	
N3	0.52395 (13)	0.13463 (14)	0.34360 (11)	0.0189 (3)	
C3	0.66330 (16)	0.21100 (17)	0.38107 (13)	0.0184 (3)	
C4	0.49578 (15)	−0.04876 (17)	0.31667 (13)	0.0222 (3)	
H6	0.4772	−0.0674	0.2228	0.027*	
H7	0.4040	−0.0858	0.3545	0.027*	
H8	0.5876	−0.1142	0.3549	0.027*	

### Atomic displacement parameters (Å^2^)

**Table:**

	*U*^11^	*U*^22^	*U*^33^	*U*^12^	*U*^13^	*U*^23^
Cl1	0.0170 (2)	0.0197 (2)	0.0238 (2)	−0.00117 (10)	0.00001 (14)	−0.00029 (10)
O1	0.0308 (6)	0.0202 (6)	0.0562 (7)	0.0037 (4)	0.0058 (5)	0.0023 (5)
N1	0.0176 (6)	0.0207 (6)	0.0312 (6)	−0.0022 (4)	−0.0029 (5)	−0.0001 (5)
N2	0.0203 (6)	0.0182 (6)	0.0262 (6)	−0.0030 (4)	0.0019 (5)	−0.0020 (4)
C1	0.0225 (7)	0.0205 (7)	0.0279 (7)	0.0009 (5)	0.0055 (5)	0.0006 (5)
C2	0.0162 (7)	0.0191 (6)	0.0276 (7)	0.0028 (5)	0.0021 (5)	0.0011 (6)
N3	0.0166 (5)	0.0163 (6)	0.0233 (6)	−0.0002 (4)	0.0007 (4)	0.0001 (4)
C3	0.0203 (7)	0.0188 (7)	0.0163 (6)	−0.0019 (5)	0.0032 (5)	−0.0007 (4)
C4	0.0195 (7)	0.0175 (6)	0.0285 (7)	−0.0017 (5)	0.0002 (5)	−0.0008 (5)

### Geometric parameters (Å, º)

**Table:**

O1—C1	1.1976 (18)	C2—N3	1.4533 (16)
N1—C3	1.3094 (18)	C2—H4	0.9700
N1—H1	0.8600	C2—H5	0.9700
N1—H2	0.8600	N3—C3	1.3237 (18)
N2—C3	1.3647 (17)	N3—C4	1.4530 (17)
N2—C1	1.3856 (17)	C4—H6	0.9600
N2—H3	0.8921	C4—H7	0.9600
C1—C2	1.5118 (19)	C4—H8	0.9600
			
C3—N1—H1	120.0	H4—C2—H5	109.2
C3—N1—H2	120.0	C3—N3—C4	127.01 (11)
H1—N1—H2	120.0	C3—N3—C2	110.53 (11)
C3—N2—C1	110.62 (11)	C4—N3—C2	121.15 (10)
C3—N2—H3	126.1	N1—C3—N3	126.05 (12)
C1—N2—H3	123.3	N1—C3—N2	123.57 (12)
O1—C1—N2	126.44 (13)	N3—C3—N2	110.36 (11)
O1—C1—C2	127.82 (12)	N3—C4—H6	109.5
N2—C1—C2	105.73 (11)	N3—C4—H7	109.5
N3—C2—C1	102.59 (11)	H6—C4—H7	109.5
N3—C2—H4	111.2	N3—C4—H8	109.5
C1—C2—H4	111.2	H6—C4—H8	109.5
N3—C2—H5	111.2	H7—C4—H8	109.5
C1—C2—H5	111.2		

### Hydrogen-bond geometry (Å, º)

**Table:**

*D*—H···*A*	*D*—H	H···*A*	*D*···*A*	*D*—H···*A*
N1—H1···Cl1^i^	0.86	2.42	3.2714 (12)	169
N1—H2···Cl1^ii^	0.86	2.32	3.1506 (12)	163
N2—H3···Cl1	0.89	2.31	3.1808 (11)	165
C2—H4···Cl1^iii^	0.97	2.69	3.6271 (14)	162
C4—H8···Cl1^i^	0.96	2.77	3.7241 (13)	175

Symmetry codes: (i) *x*, *y*−1, *z*; (ii) −*x*+2, −*y*+1, −*z*+1; (iii) −*x*+1, −*y*+1, −*z*+1.
